# Development and validation of a cytokine-based diagnostic model for Kikuchi–Fujimoto disease

**DOI:** 10.3389/fimmu.2026.1701510

**Published:** 2026-07-27

**Authors:** Xiaona Zhu, Chen Liu, Zhi Yang, Yanyan Huang, Ying Luo, Jun Yang, Tingyan He

**Affiliations:** 1 Department of Rheumatology and Immunology, Shenzhen Children’s Hospital Affiliated to Shenzhen University, Shenzhen, China; 2 Department of Biostatistics and Health Data Science, University of Pittsburgh, Pittsburgh, PA, United States

**Keywords:** cytokines, IFN-γ, IFN-γ/IL-6 ratio, interleukin-10, Kikuchi-Fujimoto disease

## Abstract

**Objective:**

The diagnosis of Kikuchi–Fujimoto disease (KFD) is challenging, which is mainly based on typical histopathological findings from a lymph node biopsy. We aimed to develop and validate a predictive model for the diagnosis of KFD based on cytokine profiles and other laboratory findings.

**Methods:**

This study included pediatric patients with KFD and controls with other febrile diseases in a retrospective training cohort (*n* = 673) and a prospective validation cohort (*n* = 116). Clinical data, laboratory parameters, and serum cytokines were collected. Variables with *P* < 0.05 in univariate analysis and acceptable collinearity were entered into multivariable logistic regression with stepwise selection. A nomogram was constructed based on the final model. Model performance was evaluated using ROC analysis, calibration curves, Hosmer–Lemeshow test, Brier score, and decision curve analysis.

**Results:**

The final model included age, WBC, neutrophil, hemoglobin, platelets, ESR, IL-10, and IFN-γ/IL-6, with IFN-γ/IL-6 modeled as a 4-knot restricted cubic spline. The derived nomogram showed excellent performance in the training cohort (AUC = 0.989, sensitivity 97.5%, specificity 94.4%) and maintained robust discrimination in the external validation cohort (AUC = 0.971, sensitivity 94.1%, specificity 93.9%). Calibration was good in both cohorts (Hosmer–Lemeshow *P* = 0.99 and 0.19, external calibration slope 0.88), with low Brier scores (0.025 and 0.060). The model achieved high predictive accuracy, with 0.966 (*κ* = 0.836) in the training cohort and 0.931 (*κ* = 0.831) in the external cohort.

**Conclusions:**

This study proposes the first validated diagnostic model for KFD, demonstrating excellent performance and highlighting the IFN-γ/IL-6 ratio as a key predictor. With further validation, it may serve as a practical decision-support tool to guide clinical management.

## Introduction

Kikuchi–Fujimoto disease (KFD) is a benign, self-limiting inflammatory disease characterized by fever and cervical lymphadenopathy. The clinical presentation of KFD is highly non-specific: patients typically present with lymphadenopathy, fever, night sweats, weight loss, myalgia, and fatigue (1–3). Laboratory findings may include leukopenia, elevated erythrocyte sedimentation rate, and abnormal liver function tests, while imaging features are similarly non-specific (1, 4, 5). As a result, the diagnosis of KFD is challenging and frequently mistaken for a wide range of conditions that share similar clinical features, leading to diagnostic delays and unnecessary interventions (4, 5).

The definitive diagnosis of KFD currently relies on histopathological examination of an excisional lymph node biopsy (6, 7). The affected lymph nodes show patchy necrosis, karyorrhectic debris, and infiltration by histiocytes and CD8^+^ cytotoxic T cells (6, 7). However, lymph node biopsy is invasive and may be associated with overlapping histopathological features with other diseases, such as lupus lymphadenitis and lymphoma (8–10). Therefore, a non-invasive diagnostic approach that may greatly help to differentiate KFD from other conditions presenting with fever and lymphadenopathy is highly desirable.

In recent years, serum cytokines have emerged as possibly promising biomarkers for KFD. Upregulated expression of interferon regulatory factors, interferon-induced antiviral proteins, and interferon-stimulated genes has been identified in KFD lymph node tissues, supporting a key role of the interferon pathway in its pathogenesis (11). Our previous study further demonstrated that KFD is characterized by a unique serum cytokine profile dominated by a significantly elevated interferon-γ (IFN-γ) level and a high IFN-γ/interleukin (IL)-6 ratio, which allows for the differentiation of KFD from systemic juvenile idiopathic arthritis (sJIA) and Kawasaki disease (KD) (12). Specifically, serum IFN-γ > 8.56 pg/mL and an IFN-γ/IL-6 ratio > 0.45 may highly suggest the diagnosis of KFD, while serum IL-6 > 50.45 pg/mL indicates that the probability of KFD is low and other inflammatory conditions should be prioritized (12).

Despite these promising findings, differences in serum cytokine profiles and other laboratory parameters between KFD and other common differential diagnoses remain incompletely characterized. It is unclear whether distinct cytokine patterns can reliably distinguish KFD from a broader spectrum of inflammatory diseases presenting with fever and lymphadenopathy, including autoimmune disorders, infectious lymphadenitis, and other systemic inflammatory conditions. Further exploration of these differences is crucial to promote early diagnosis, avoid unnecessary invasive procedures, and optimize clinical management.

Therefore, in this study, we aimed to develop and validate a diagnostic nomogram incorporating clinical features and serum cytokine levels for differentiating KFD from other diseases presenting with fever and lymphadenopathy, and to provide a practical tool and non-invasive tool for clinicians.

## Materials and methods

### Study participants

**Figure 1:**
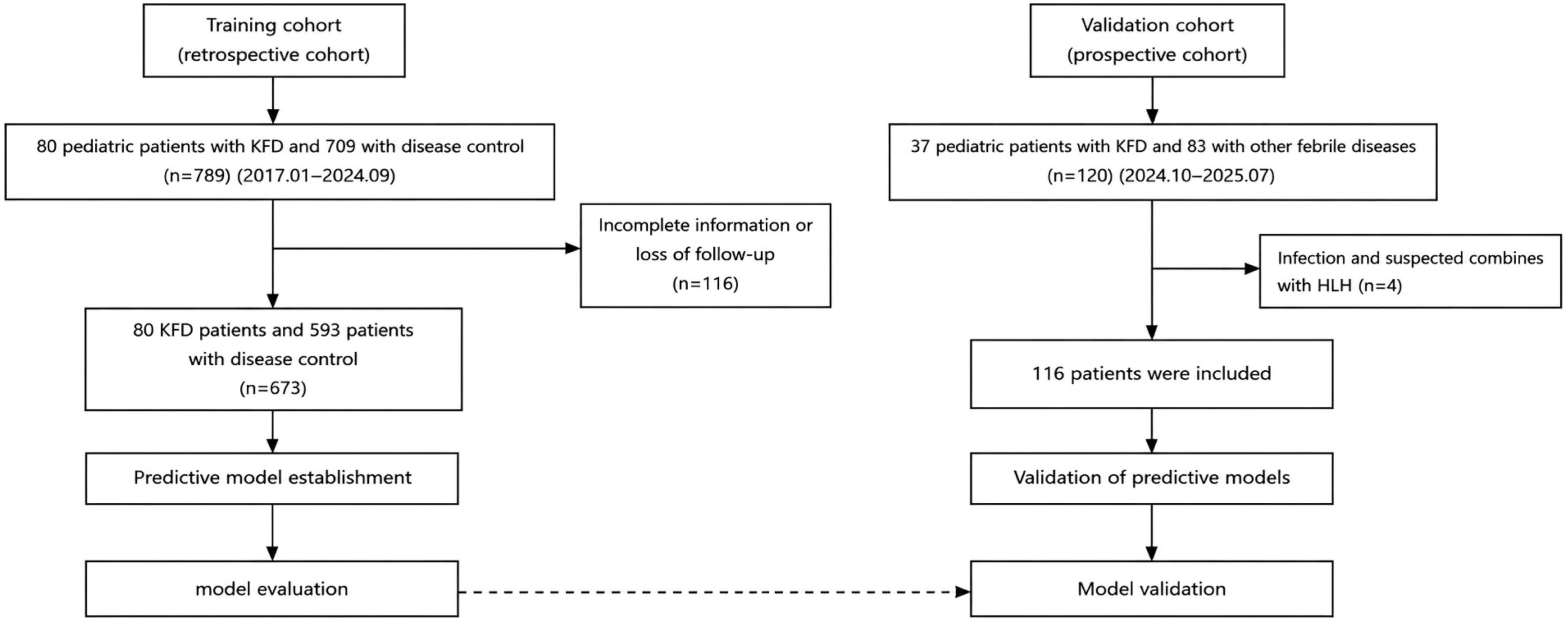
Flowchart of this research. KFD, Kikuchi–Fujimoto disease.

Study participants comprised the observation group with KFD and the diseased controls with other febrile diseases. Two distinct cohorts were established: a retrospective training cohort, which included 673 patients enrolled from January 2017 to September 2024 (80 with KFD and 593 disease controls), and a prospective external validation cohort, which included 116 patients recruited from October 2024 to July 2025 (34 with KFD and 82 disease controls) (**Figure 1**). The retrospective cohort was used for model development, whereas the prospective cohort served for external validation.

### Inclusion and exclusion criteria

The inclusion criteria for KFD cases were as follows: 1) pediatric patients with KFD confirmed by pathological examination of lymph node biopsy (7); 2) absence of immunodeficiency, chromosomal abnormalities, malignancies, major trauma, acute infection, or other rheumatic autoimmune diseases; and 3) cytokine profiling performed during the active disease phase (fever ≥ 2 days), without concurrent acute infection and prior to the initiation of glucocorticoids or immunosuppressive therapy.

The inclusion criteria for disease controls included 1) meeting the diagnostic criteria for KD (13), sJIA (14), periodic fever syndromes (15), bacterial infection, Epstein–Barr virus (EBV) infection, malignancy, other viral infections (adenovirus, metapneumovirus, influenza A/B, parainfluenza), or other rheumatic and immune diseases, such as systemic lupus erythematosus (SLE) (**Supplementary Figure 1**); 2) cytokine profiling performed during the active disease phase (fever ≥ 2 days); and 3) no history of chemotherapy, immunosuppressants, or other relevant medications within 2 weeks before sample collection. The exclusion criteria for controls included 1) lack of cytokine profiling or assessment during the afebrile phase, 2) presence of macrophage activation syndrome or hemophagocytic lymphohistiocytosis (HLH) (16, 17), and 3) possible concurrent diagnosis of KFD.

### Data collection

Clinical data were extracted from electronic medical records, including demographic information, clinical manifestations, laboratory findings, and serum cytokine levels. Laboratory data included white blood cell count, neutrophil count, platelet count, hemoglobin, erythrocyte sedimentation rate (ESR), C-reactive protein (CRP), ferritin, and fibrinogen. Cytokines measured included IFN-γ, IL-10, IL-6, tumor necrosis factor-α (TNF-α), IL-4, and IL-2.

### Missing data handling

In the training cohort, 2 of the 80 KFD cases had missing data; due to the small sample size, mean imputation was applied. Among controls, 709 samples were initially screened; 116 with incomplete data were excluded, leaving 593 samples for analysis.

### Ethics

The study was conducted in compliance with the ethical standards described in the Declaration of Helsinki. Ethical approval was obtained from the Ethics Committee of Shenzhen Children’s Hospital (Approval No. 202407002). The parents or legal guardians of participants provided written informed consent to participate in this study.

### Statistical analysis

Statistical analyses were performed using SPSS Statistics version 26.0 (IBM Corp., Armonk, NY, USA) and R version 4.4.0 (R Foundation for Statistical Computing, Vienna, Austria). Continuous variables were assessed for normality using the Shapiro–Wilk test. As none followed a normal distribution, they were presented as median (interquartile range) [M (P25, P75)] and compared between groups using the Wilcoxon rank-sum test. Categorical variables were expressed as frequency and percentage [*n* (%)] and compared using the *χ*^2^ test. Model fitting and validation used the following R packages: rms (v8.0.0) for the restricted cubic spline model, nomogram, and bootstrap internal validation and calibration; pROC (v1.19.0.1) for ROC and AUC; ResourceSelection (v0.3.6) for the Hosmer–Lemeshow test; caret (v7.0.1) for classification accuracy and kappa; and dcurves (v0.5.1) for decision curve analysis.

### Model development and nomogram construction

Variables with *P* < 0.05 in univariate analyses were assessed for multicollinearity using linear regression. Variance inflation factor (VIF) < 10 and tolerance > 0.1 were considered acceptable. Eligible variables were entered into multivariable logistic regression using stepwise selection. Based on the final regression model, a nomogram was constructed. The IFN-γ/IL-6 ratio was modeled using a 4-knot restricted cubic spline (RCS) with knots placed at the 5th, 35th, 65th, and 95th percentiles. Because ESR, IL-10, and the IFN-γ/IL-6 ratio were markedly right-skewed (skewness range: 5.0–9.9), we conducted sensitivity analyses using a linear model, a log-transformed model for the IFN-γ/IL-6 ratio, log-transformed models for all three variables, and a three-variable restricted cubic spline model. The full results of the sensitivity models are provided in **Supplementary Tables 2, 3**.

### Model validation

Model performance was evaluated using receiver operating characteristic (ROC) curve analysis for discrimination, the Hosmer–Lemeshow (H-L) goodness-of-fit test, calibration plots, and Brier scores for calibration, and decision curve analysis (DCA) for clinical utility. All statistical tests were two-sided, and *P* < 0.05 was considered statistically significant. Internal validation used 1,000 bootstrap resamples to obtain optimism-corrected discrimination and calibration. The model was then applied to the external cohort with its training coefficients fixed (no refitting). Predicted probabilities and serum IFN-γ, IL-6, and the IFN-γ/IL-6 ratio were compared across diagnostic subgroups to assess discrimination against challenging mimics.

## Results

### Comparison of clinical and laboratory parameters between KFD and control groups

Compared with disease controls, patients with KFD had a significantly older age at disease onset (median 9.80 vs. 4.00 years, *P* < 0.001). A higher proportion of KFD patients presented with cervical lymphadenopathy [80 (100%) vs. 336 (56.7%), *P* < 0.001] and splenomegaly [12 (15.0%) vs. 40 (6.8%), *P* = 0.009], whereas skin rash [9 (11.3%) vs. 278 (46.9%), *P* < 0.001] and arthralgia [2 (2.5%) vs. 83 (14.0%), *P* = 0.004] were less common. KFD patients exhibited lower white blood cell counts, neutrophil counts, and platelet counts (all *P* < 0.001), along with reduced levels of CRP, ESR, fibrinogen, serum IL-10, and IL-6 (all *P* < 0.001). In contrast, hemoglobin levels, serum IFN-γ, AST, and ferritin were significantly increased in the KFD group (all *P* < 0.001), and the IFN-γ/IL-6 ratio was markedly higher (2.63 vs. 0.12, *P* < 0.001). No significant differences were observed between the two groups in ALT, serum IL-2, or IL-4 levels (**Table 1**).

**Table 1:** Comparison of clinical and laboratory parameters in the two groups.

Variables	KFD (n = 80)	Disease controls (n = 593)	z/χ^2^	P-value
Gender (female/male)	21/59	235/358	5.353	0.021
Age at disease onset (years)	9.80 (7.47, 12.15)	4.00 (2.00, 7.10)	−9.883	**<0.001**
Rash, n (%)	9 (11.25)	278 (46.88)	36.587	**<0.001**
Cervical lymphadenopathy, n (%)	80 (100.00)	336 (56.66)	56.091	**<0.001**
Arthralgia, n (%)	2 (2.50)	83 (14.00)	8.443	**0.004**
Hepatomegaly, n (%)	21 (26.25)	111 (18.72)	2.536	0.111
Splenomegaly, n (%)	12 (15.00)	40 (6.75)	6.737	**0.009**
White blood cell (×109/L)	3.43 (2.70, 4.68)	12.18 (8.20, 16.40)	−12.5	**<0.001**
Neutrophils (×109/L)	1.56 (1.07, 2.49)	7.40 (4.70, 11.70)	−12.022	**<0.001**
Hemoglobin (g/L)	117.50 (110.75, 124.00)	111.00 (103.00, 118.00)	−4.179	**<0.001**
Platelets (×109/L)	202.00 (172.75, 255.00)	302.00 (228.00, 402.00)	−7.501	**<0.001**
C-reactive protein (mg/L)	4.85 (2.17, 11.69)	42.80 (16.40, 78.90)	−10.5	**<0.001**
ESR (mm/h)	26.50 (17.75, 38.25)	44.00 (25.00, 65.00)	−5.543	**<0.001**
ALT (U/mL)	17.50 (12.75, 37.50)	16.00 (11.00, 32.00)	−1.286	**0.198**
AST (U/mL)	35.00 (27.75, 50.50)	28.00 (23.00, 40.00)	−3.608	**<0.001**
Ferritin (ng/mL)	332.00 (217.82, 526.65)	196.00 (124.00, 332.94)	−5.312	**<0.001**
Fibrinogen (g/L)	3.75 (3.35, 4.23)	4.88 (3.83, 6.43)	−7.071	**<0.001**
Interleukin-2 (pg/mL)	0.80 (0.17, 2.06)	1.06 (0.26, 2.26)	−1.251	0.211
Interleukin-4 (pg/mL)	1.81 (0.95, 2.68)	1.73 (1.11, 2.85)	−0.654	0.513
Interleukin-6 (pg/mL)	6.81 (4.09, 13.17)	31.62 (13.68, 76.46)	−9.297	**<0.001**
Interleukin-10 (pg/mL)	3.17 (1.89, 4.92)	4.87 (2.53, 11.78)	−4.247	**<0.001**
Tumor necrosis factor-α (pg/mL)	1.77 (0.71, 2.85)	2.25 (0.89, 3.59)	−2.367	**0.018**
Interferon-γ (pg/mL)	19.39 (10.21, 34.77)	3.06 (1.73, 7.64)	−9.911	**<0.001**
IFN-γ/IL-6	2.63 (1.43, 4.79)	0.12 (0.04, 0.43)	−13.226	**<0.001**

KFD, Kikuchi–Fujimoto disease; ESR, erythrocyte sedimentation rate; ALT, alanine transaminase; AST, aspartate aminotransferase; IFN-γ, interferon-γ; IL-6, interleukin-6.

Bold values indicates statistical significance.

### Univariable and multivariable logistic regression analyses

Among the 18 continuous variables in **Table 1**, 12 parameters were selected based on the results of univariate analysis, collinearity analysis, and clinical relevance for further multivariable logistic regression, including age, WBC, neutrophil, hemoglobin, platelets, CRP, ESR, AST, FER, Fib, IL-10, and IFN-γ/IL-6 (**Table 2**). The final multivariable logistic regression model included age, WBC, neutrophil, hemoglobin, platelets, ESR, IL-10, and IFN-γ/IL-6 with RCS (**Table 3**). The regression equation is shown below:

**Table 2:** Collinearity diagnostics of different variables.

Variables	Tolerance	VIF
Age (years)	0.662	1.51
White blood cell (×109/L)	0.112	8.963
Neutrophils (×109/L)	0.113	8.888
Hemoglobin (g/L)	0.798	1.253
Platelets (×109/L)	0.587	1.703
C-reactive protein (mg/L)	0.512	1.952
ESR (mm/h)	0.582	1.719
AST (U/mL)	0.805	1.242
Ferritin (ng/mL)	0.748	1.338
Fibrinogen (g/L)	0.411	2.432
Interleukin-10 (pg/mL)	0.921	1.086
IFN-γ/IL-6	0.819	1.221

VIF, variance inflation Factor; ESR, erythrocyte sedimentation rate; AST, aspartate aminotransferase; IFN-γ, interferon-γ; IL-6, interleukin-6.

**Table 3:** Coefficients of the final model.

Variable	β	OR	CI	P
Age	0.184	1.202	1.030–1.402	0.0192
WBC	−0.37	0.691	0.455–1.049	0.0829
N	−0.711	0.491	0.277–0.870	0.0149
Hb	0.07	1.072	1.032–1.113	0.000297
PLT	0.008	1.008	1.002–1.015	0.0151
ESR	0.041	1.042	1.010–1.074	0.00881
IL-10	−0.036	0.965	0.940–0.990	0.0072
IFN-γ/IL-6	Overall Wald χ^2^ = 38.82 (df = 3), P < 0.001; non-linear χ^2^ = 21.39, P < 0.001			

*β*, regression coefficients; Cl, confidence interval; WBC, white blood cell; N, neutrophil; Hb, hemoglobin; PLT, platelets; ESR, erythrocyte sedimentation rate; IL-10, interleukin-10.

Logit(P) = −26.424 + 0.184 × age − 0.370 × WBC (×10^9^/L) − 0.711 × neutrophil (×10^9^/L) + 0.070 × hemoglobin (g/L) + 0.008 × platelets (×10^9^/L) + 0.041 × ESR (mm/h) − 0.036 × IL-10 (pg/mL) + s(IFN-γ/IL-6).

where s(IFN-γ/IL-6) denotes the fitted 4-knot RCS of the IFN-γ/IL-6 ratio (knots at the 5th, 35th, 65th, and 95th percentiles: 0.011, 0.082, 0.410, and 3.554), with the exact spline basis coefficients given in **Supplementary Table 4**.

### Development and ROC evaluation of the nomogram model

**Figure 2:**
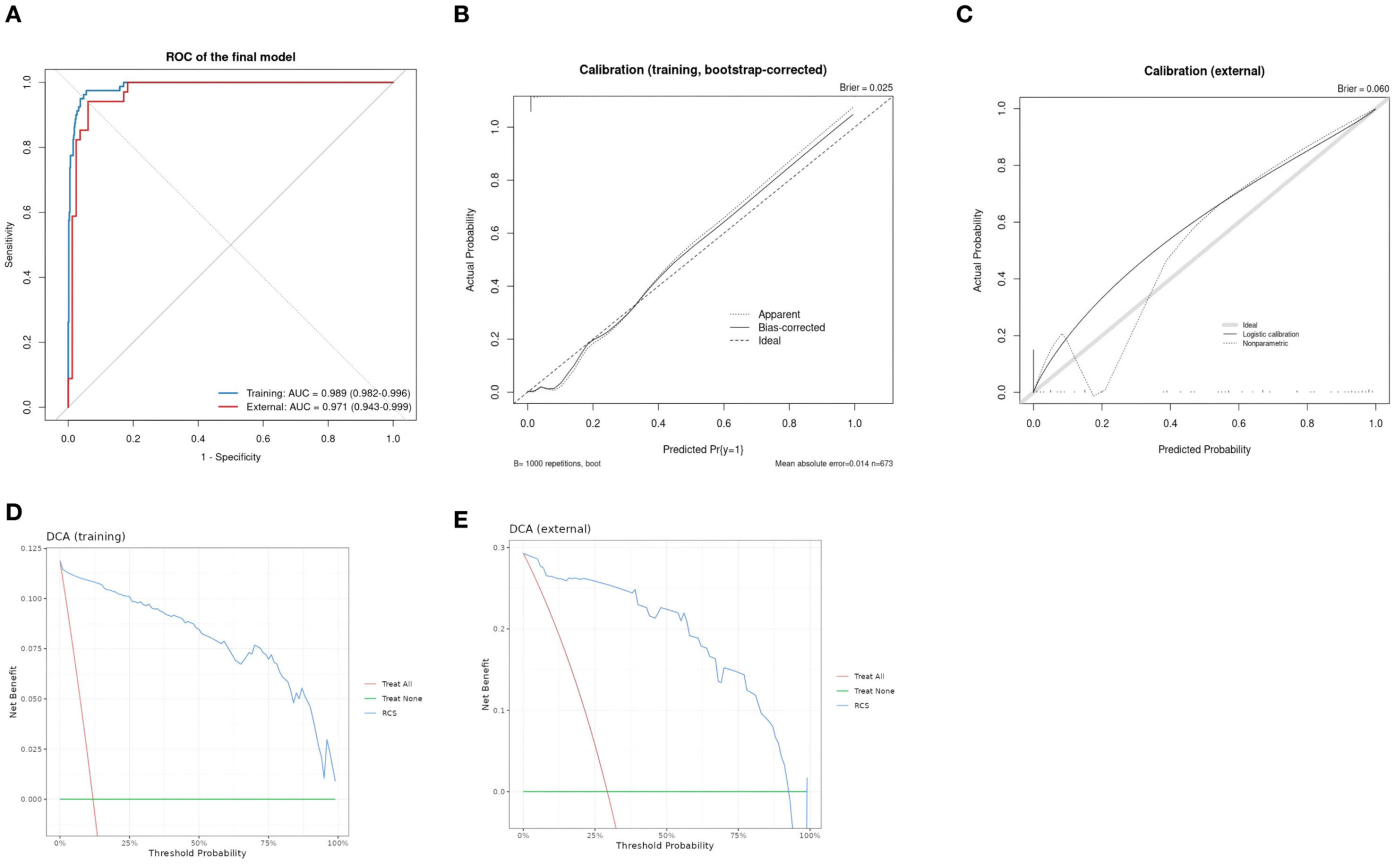
Evaluation of the nomogram model. ROC curve of the final model in the training cohort (blue) and the external validation cohort (red) **(A)**. Calibration curve of the nomogram model in the training cohort **(B)** and the external validation cohort **(C)** based on the calibration plot and Brier score. The *x*-axis shows the predicted probability of KFD, and the *y*-axis shows the observed probability of KFD. The apparent line indicates the actual performance of the prediction model. The bias-corrected line indicates the performance of the prediction model after the correction of the overfitting situation. DCA curve of the nomogram model in the training cohort **(D)** and the external validation cohort **(E)**. The *x*-axis displays the probability threshold. The *y*-axis indicates the degree of benefit to which the patient was diagnosed with KFD. AUC, the area under the curve; DCA, decision curve analysis; ROC, receiver operating characteristic.

A nomogram prediction model in the training cohort was subsequently constructed based on the final prediction model for the diagnosis of KFD (**Supplementary Figure 1**). The AUC of the ROC curve was 0.989 (95% CI: 0.982~0.996) in the training cohort. The maximum value of the Youden index was 0.919, and the optimal cutoff value was 0.157 with a sensitivity of 97.5% and a specificity of 94.4% (**Table 4**; **Figure 2A**).

**Table 4:** Receiver operating characteristic curve analysis of both cohorts.

Cohort	AUC	CI	Cutoff	Sensitivity (%)	Specificity (%)	Youden index	Accuracy	Kappa
Training (*n* = 673)	0.989	0.982–0.996	0.157	97.5	94.4	0.919	0.966	0.836
External (*n* = 116)	0.971	0.943–0.999	0.388	94.1	93.9	0.88	0.931	0.831

AUC, area under the ROC curve; CI, confidence interval.

In the external validation cohort, the AUC of the ROC curve was 0.971 (95% CI: 0.943~0.999). The maximum value of the Youden index was 0.880, and the optimal cutoff value was 0.388, with a sensitivity of 94.1% and a specificity of 93.9% (**Table 4**; **Figure 2A**).

### Evaluation of the nomogram model using the H-L test, calibration plot, and Brier scores

In the training cohort, the Hosmer–Lemeshow test indicated a good model fit (*χ*^2^ = 1.67, *P* = 0.99), and the calibration curve for the nomogram in the training cohort showed a strong alignment between the calibration and standard curves (**Figure 2B**). Furthermore, the model’s low Brier score (0.025) further affirmed its good overall predictive accuracy (**Figure 2B**).

In the external validation cohort, the Hosmer–Lemeshow test indicated a good model fit (*χ*^2^ = 11.22, *P* = 0.19). The calibration curve for the nomogram in the external cohort showed a good alignment between the calibration and standard curves (**Figure 2C**). The model’s Brier score was 0.060, further confirming its good overall predictive accuracy (**Figure 2C**).

### DCA evaluation of the nomogram model

In the training cohort, the DCA demonstrated that using the nomogram model can significantly enhance clinical benefits (**Figure 2D**). Internal validation showed that the predictive model had a good predictive value with an accuracy of 0.966 and a kappa value of 0.836.

In the external validation cohort, the DCA also demonstrated that using the nomogram model can significantly enhance clinical benefits (**Figure 2E**). The predictive accuracy in the external validation cohort was 0.931 with a kappa value of 0.831.

### Discrimination of KFD from individual differential diagnoses

In the external cohort, at the training-derived cutoff of 0.157, the median predicted probability of KFD was 0.82, versus 0.01 in SLE (*n* = 7), 0.09 in malignancy (*n* = 2) and ≤0.00 in bacterial infection (*n* = 9), viral/EBV infection (*n* = 14), KD (*n* = 17), sJIA (*n* = 7), and PFAPA (*n* = 10) (**Supplementary Table 1**). KFD had the highest IFN-γ (16.14 pg/mL) and the lowest IL-6 (12.71 pg/mL) and, thus, the highest IFN-γ/IL-6 ratio (1.24 vs. 0.03–0.42). Pairwise discrimination was high for every differential diagnosis (AUC 0.975 vs. SLE, 0.971 vs. malignancy, 1.000 vs. sJIA, 0.996 vs. viral/EBV, 1.000 vs. KD, 0.988 vs. PFAPA), and excluding bacterial controls barely changed overall discrimination (AUC 0.971 to 0.968).

## Discussion

This study successfully developed and validated a multivariable logistic regression model to predict the risk of KFD. To our knowledge, this is the first model of its kind that integrates age, WBC, neutrophil, hemoglobin, platelets, ESR, IL-10, and IFN-γ/IL-6 into a clinically applicable risk score. The model demonstrated excellent performance in the training cohort (AUC = 0.989, 95% CI: 0.982~0.996) and retained good discrimination and calibration in an independent external validation cohort (AUC = 0.971, 95% CI: 0.943~0.999). By combining conventional hematological parameters with cytokine-based immunological markers, this model provides a quantitative and integrative diagnostic approach that reflects both systemic inflammation and immune dysregulation in KFD.

Previous studies have predominantly described the clinical manifestations of KFD, such as fever, cervical lymphadenopathy, and constitutional symptoms, or reported isolated laboratory abnormalities, including leukopenia and elevated ESR (18, 19). While these features may help raise suspicion, their diagnostic specificity is limited, and lymph node biopsy has remained the gold standard for confirmation. In contrast, our model incorporated multiple laboratory and immune-related variables, thereby improving discriminatory ability. Notably, the inclusion of IL-10 and the IFN-γ/IL-6 ratio emphasizes the contribution of cytokine imbalance in KFD pathogenesis (11). This integrative framework provides a stronger diagnostic value than reliance on clinical features alone.

The final model identified IFN-γ/IL-6 as the strongest independent predictor (overall Wald *χ*^2^ = 38.8, *P* < 0.001), modeled as a restricted cubic spline with a significant non-linear component (*P* < 0.001). In the linear sensitivity model, this ratio carried the largest effect (OR = 2.748, 95% CI: 1.942~3.890). Previous studies have reported elevated serum IFN-γ and IL-6 levels during the acute phase of KFD (11, 12). Our earlier work demonstrated that patients with KFD showed mildly elevated IL-6 but significantly increased IFN-γ levels, leading to a markedly higher IFN-γ/IL-6 ratio compared with sJIA or KD (12). In this study, the disease control group was expanded to include a broad spectrum of febrile conditions, such as KD, sJIA, periodic fever syndromes, bacterial infections, EBV infection, malignancies, other infections, and other rheumatic or autoimmune diseases. Across these comparisons, patients with KFD consistently exhibited lower serum IL-6 levels and higher IFN-γ levels. Further pairwise discrimination analysis indicated that the ratio reflects genuinely elevated IFN-γ rather than high IL-6 in bacterial controls, reinforcing the biological plausibility of the IFN-γ/IL-6 ratio as a robust diagnostic marker.

Despite the strengths of this study, several limitations should be acknowledged. First, the sample size of KFD was relatively limited. Second, both model construction and external validation were conducted in single-center cohorts only in the Chinese population, possibly limiting the generalizability of our findings. Third, cytokine assays such as IL-10 and IFN-γ/IL-6 are not yet widely available in routine clinical practice, which may temporarily limit immediate clinical application. Fourth, although controls were required to have lymphadenopathy, cervical lymphadenopathy was not mandatory and a considerable proportion of controls had bacterial infections; however, sensitivity analyses restricting controls to those with cervical lymphadenopathy and excluding bacterial infection controls showed essentially unchanged discrimination, indicating that these factors did not materially inflate model performance. Nevertheless, these limitations do not undermine the main findings; the proposed nomogram demonstrates robust diagnostic performance and offers practical clinical utility in differentiating KFD from other inflammatory diseases, especially when used in conjunction with clinical presentation. Future multicenter studies with larger sample sizes and stricter control design are still warranted to further validate and extend our conclusions. In conclusion, this study demonstrates that the IFN-γ/IL-6 ratio is a powerful biomarker for distinguishing KFD from other inflammatory conditions with fever. The proposed diagnostic model incorporating this ratio achieved high accuracy in both derivation and validation cohorts.

## Conclusions

In summary, we present the first multivariable logistic regression model for the diagnosis of KFD, integrating routine hematological and inflammatory parameters with immune-based cytokine markers. The model demonstrated excellent diagnostic performance and identified IFN-γ/IL-6 as the strongest independent predictor. With further validation and refinement, this model may be transformed into practical clinical tools to assist in the timely recognition and management of KFD.

## Data Availability

The raw data supporting the conclusions of this article will be made available by the authors, without undue reservation.

## Supplementary material

Supplementary Figure 1 Nomogram of the final model for the diagnosis of KFD in the training cohort WBC, white blood cell; N, neutrophil; Hb, hemoglobin; PLT, platelets; ESR, erythrocyte sedimentation rate; IL10, interleukin-10.

Supplementary Figure 2 Restricted cubic spline effect of IFN-γ/IL-6 WBC, white blood cell; N, neutrophil; Hb, hemoglobin; PLT, platelets; ESR, erythrocyte sedimentation rate; IL10, interleukin-10.
